# Effects of scaling up various community-level interventions on child mortality in Burundi, Kenya, Rwanda, Uganda and Tanzania: a modeling study

**DOI:** 10.1186/s41256-019-0106-2

**Published:** 2019-05-29

**Authors:** Celestin Hategeka, Germaine Tuyisenge, Christian Bayingana, Lisine Tuyisenge

**Affiliations:** 1 Centre for Health Services and Policy Research, School of Population and Public Health, Faculty of Medicine, University of British Columbia, Vancouver, BC Canada; 2 Collaboration for Outcomes Research and Evaluation, Faculty of Pharmaceutical Sciences, University of British Columbia, Vancouver, BC Canada; 3 Department of Geography, Simon Fraser University, Burnaby, BC Canada; 4 Bloomberg School of Public Health, Johns Hopkins Hospital, Baltimore, MD USA; 5 Department of Paediatrics and Child Health, University Teaching Hospital of Kigali, Kigali, Rwanda

**Keywords:** Global health, Child health, Community level interventions, Community health workers, East African community

## Abstract

**Background:**

Improving child health remains one of the most significant health challenges in sub-Saharan Africa, a region that accounts for half of the global burden of under-five mortality despite having approximately 13% of the world population and 25% of births globally. Improving access to evidence-based community-level interventions has increasingly been advocated to contribute to reducing child mortality and, thus, help low-and middle-income countries (LMICs) achieve the child health related Sustainable Development Goal (SDG) target. Nevertheless, the coverage of community-level interventions remains suboptimal. In this study, we estimated the potential impact of scaling up various community-level interventions on child mortality in five East African Community (EAC) countries (i.e., Burundi, Kenya, Rwanda, Uganda and the United Republic of Tanzania).

**Methods:**

We identified ten preventive and curative community-level interventions that have been reported to reduce child mortality: Breastfeeding promotion, complementary feeding, vitamin A supplementation, Zinc for treatment of diarrhea, hand washing with soap, hygienic disposal of children’s stools, oral rehydration solution (ORS), oral antibiotics for treatment of pneumonia, treatment for moderate acute malnutrition (MAM), and prevention of malaria using insecticide-treated nets and indoor residual spraying (ITN/IRS). Using the Lives Saved Tool, we modeled the impact on child mortality of scaling up these 10 interventions from baseline coverage (2016) to ideal coverage (99%) by 2030 (ideal scale-up scenario) relative to business as usual (BAU) scenario (forecasted coverage based on prior coverage trends). Our outcome measures include number of child deaths prevented.

**Results:**

Compared to BAU scenario, ideal scale-up of the 10 interventions could prevent approximately 74,200 (sensitivity bounds 59,068–88,611) child deaths by 2030 including 10,100 (8210–11,870) deaths in Burundi, 10,300 (7831–12,619) deaths in Kenya, 4350 (3678–4958) deaths in Rwanda, 20,600 (16049–25,162) deaths in Uganda, and 28,900 (23300–34,002) deaths in the United Republic of Tanzania. The top four interventions (oral antibiotics for pneumonia, ORS, hand washing with soap, and treatment for MAM) account for over 75.0% of all deaths prevented in each EAC country: 78.4% in Burundi, 76.0% in Kenya, 81.8% in Rwanda, 91.0% in Uganda and 88.5% in the United Republic of Tanzania.

**Conclusions:**

Scaling up interventions that can be delivered at community level by community health workers could contribute to substantial reduction of child mortality in EAC and could help the EAC region achieve child health-related SDG target. Our findings suggest that the top four community-level interventions could account for more than three-quarters of all deaths prevented across EAC countries. Going forward, costs of scaling up each intervention will be estimated to guide policy decisions including health resource allocations in EAC countries.

## Introduction

Under-five mortality rate (the probability of a child dying between birth and exactly five years of age) reduced worldwide by approximately 53% from 1990 to 2015 (from 91 deaths per 1000 live births in 1990 to 41 in 2015), with a faster reduction starting in 2000 due to the substantial efforts in the Millennium Development Goals (MDGs) era; the annual rate of reduction in under-5 mortality has increased from 1.9% before the year 2000 to 4% between 2000 and 2015 [1–4]. Although all regions have halved their under-five mortality rates in the same time period, disparities in mortality across and within regions remain and hence the continued efforts to reduce child mortality further are imperative.

Improving child survival remains one of the most significant health challenges in sub-Saharan Africa (SSA), a region that accounts for half of the global burden of under-five mortality despite having approximately 13% of the world population and 25% of births globally [5]. About 73% of under-five deaths occurred in two regions in 2016: Africa (48%) and South-East Asia (25%). The highest under-five mortality rate is in the African region (76.5 per 1000 live births), and the lowest in Europe (9.6 per 1000 live births) [3]. The fourth MDG which aimed to reduce the under-five mortality by 2/3 between 1990 and 2015 was not achieved by many countries, suggesting that many children are still at risk of dying before their fifth year of life [6].

To build on the efforts put forth during the MDGs era, the Sustainable Development Goals (SDGs) ushered in a call to end preventable newborns and children deaths by 2030, with all countries aiming to reduce neonatal mortality to at least as low as 12 per 1000 live births and under-five mortality to at least as low as 25 per 1000 live births. To track progress better, the SDGs emphasize the need for reliable and disaggregated data by multiple criteria (for example, socio-demographic characteristics) which could have an impact on child health outcomes [7]. Identifying the location and characteristics of the most at-risk children is of paramount importance to addressing under-five mortality in the SDG era (2016–2030). Endeavors to this end will help highlight how sustainable development efforts could benefit various populations across different countries.

As seen in the MDGs era, achieving child mortality reduction targets has been a considerable challenge and, in some countries, significant progress was made possible by innovative programs that were introduced in the health systems. For example, the introduction of community level interventions (CLIs) delivered by community health workers (CHWs) helped bridge the shortage in healthcare workers and improve access to health services among hard-to-reach populations, especially in SSA [8]. Understanding and estimating the potential impacts of CLIs on reducing child mortality is the focus of this study. Integration of community-level interventions and scaling up evidence-based CHWs programs may give a much-needed push to achieve SDGs efforts to reduce preventable child deaths.

Introduced in 1960s, the role of CHWs has been recognized as part of the integral health system as a response to linking communities to the formal health system [9]. CHWs are defined, according to Lewin et al. (2010), as any lay health workers who live in the area they serve, are primarily based in the community where they serve (as opposed to a health facility), belong to the formal health system (i.e., they are managed by the government or an implementing NGO), perform tasks related to health care delivery, and have received organized training but may not have received formal or paraprofessional certification or tertiary education degree [10, 11]. In the context of SSA, CLIs and CHW programs could be a helpful tool for many national healthcare systems where shortage of healthcare professionals continues to be problematic [12].

It has been advocated that strengthening community health systems can help reduce under-five child mortality burden in low-income settings like SSA. According to Haines et al. (2007), many life-saving child health interventions can be provided at community levels [13]. As highlighted by Lewin et al. (2010) and Christoper et al. (2011) CHWs and CLIs have been effective in promoting breastfeeding and have had a positive impact on malaria [10, 14]. Countries like Rwanda credited CHWs programs for their pivotal role in achieving MDGs 4 and 5.A [15–18]. In the year 2018, Rwanda increased the number of CHWs from 45,000 to 58,286 in order to reduce their workload and improve the quality of service they provide [19]. Where there was enough room for improvement, Rwandan community level programs were associated with a significant increase in coverage of maternal and child health services when CHWs benefited from additional support including regular training and supervision [20, 21].

Burundi ranks among the countries with high under-5 mortality worldwide with one in thirteen children at risk of dying before reaching the fifth birthday [22]. In order to improve this alarming situation in a country like Burundi, where a socio-political crisis caused the government to cut funding for healthcare by approximately 54% in 2016, strategies that focus on community health promotion using resources available in the are crucial [23]. It is in this context that CONCERN, an international NGO implemented a pilot project in Cibitoke health district, between 2014 and 2016, in order to combat key causes of under-five mortality [23]. During the pilot, 393 CHWs received training and supervision to provide treatment and health education in regard to key causes of under-five mortality. The introduction of community-based health services by community volunteers led to a coverage of 80% of population with an increase in community health services offered by CHWs in homes, an increased treatment seeking behaviour by parents/guardians and an improved knowledge for disease prevention [23].

In an effort to integrate the CHWs program into the health system and to promote community health, Uganda has started registering its existing 180,000 village health teams (VHTs) operating across the country. An additional 15,000 Community Health Extension Workers have also been recruited, hired and formally trained to provide basic primary health services at the community level across the country. VHTs have largely contributed to the improvement in access to health services in the country. A study conducted in Eastern Uganda shows that CHWs increased population coverage for maternal and child health education through home visits (83.9%), and community meetings (82.7%), from 54.8% in regions where CHWs are not fully active and health education is only offered at health facilities [24, 25].

In Tanzania, where the child mortality rate is 48.7 for every 1000 live births and there are only 0.3 doctors and 4.4 nurses and midwives for every 10,000 people, the need for primary health services closer to the communities is undeniable [26, 27]. CHWs in Tanzania are uncoordinated and unaccounted for, in terms of which regions they operate in and who funds their programs [28]. It is estimated that 41,000 CHWs are employed across the country but they are unevenly distributed across regions. Nonetheless, in regions where they received full training and support by mostly international NGOs, they have contributed greatly to the promotion of child health. For example, in regions where World Vision operates, CHWs have been trained to provide curative and preventive services for some of the major causes of child mortality including diarrhoea, pneumonia and malaria [29].

In Kenya, there are 135,000 CHWs operating across the country under the mandate of the Ministry of Health [30]. As an integral part of the health system, the country’s community-based health workers program is still undergoing scaling-up process in order to have a more sustainable CHW program model and for training, supervision and mentorship of CHWs’ services [31]. In Kenya, CHWs are mostly involved in health promotion, providing health education to families and communities; preventive care that includes the provision of mosquito nets and, curative care, including providing tables for diarrhea among under-five children. CHWs provide their services through door-to-door method or in their own homes with an operating zone of about 25 households.

An example of successful neonatal and child health CLI is found in Nepal where Female Community Health Volunteers (FCHV) have been trained to manage and treat newborn illness at the community level [32]. FCHV pay visits to newborns soon after delivery and in subsequent weeks to follow up with their wellbeing [33]. The Morang Initiative Neonatal Intervention (MINI), established between 2005 and 2009 in Morang district, documented the effectiveness of involving CHWs in the treatment of neonatal illness [34]. The MINI program identified possible severe bacterial infection in neonates and young infants and provide them with treatment with antibiotics in collaboration with facility-based CHW. As members of the community where they operate, one of FCHVs’ roles involves provided health education to the mother for the wellbeing of the baby. Results on the effectiveness of the MINI program between 2005 and 2007 showed a success of 90% in coverage [34]. In addition, the program recorded 1.5% of fatality cases compared to 5.3% in regions where the program was not implemented [34]. Ghana is another example of a successful CHW program addressing newborn and child health [32]. A study conducted in Dangme west district of Greater Accra region, Ghana highlights that parents and caregivers had little knowledge about pneumonia in under five, which in return, affected the way they understood the signs, symptoms and causes of pneumonia and their behaviour on treatment seeking [35]. However, most of the respondents (96.6%) were willing to use CHWs services for the management of pneumonia provided they were available in their communities [35]. Another study on the treatment of an all-cause mortality among under five in the same district showed a reduction in all-cause mortality of 30% among children treated by CHWs with antimalarial drugs and one of 44% for those treated with antimalarial plus an antibiotic which were provided to children in the communities, either at the CHW home or at the caregiver’s [36].

Global estimates suggest that scaling up coverage of CLIs is one of the most effective strategies to help countries achieve health related SGDs target [37]. Similarly, a South African study reports that CLIs could be cost-effective [38]. However, current national and regional estimates about potential impact of scaling up CLIs across East Africa are lacking. In this study, we estimated the potential impact of scaling up various CLIs on child mortality in five East African Community (EAC) countries (Burundi, Kenya, Rwanda, Uganda and the United Republic of Tanzania).

## Methods

### Study context

Headquartered in Arusha, Tanzania, the East African Community (EAC) is a regional intergovernmental organization bringing together Kenya, Uganda, the United Republic of Tanzania (henceforth referred to Tanzania), Burundi and Rwanda for a wider and deeper cooperation among these countries and other regional economic communities for mutual economic, social and political benefit (https://au.int/en/recs/eac). In the health sector, Yamin et al. (2017) argue that achieving universal health coverage (UHC) in EAC would require EAC countries to put in place human rights-based approaches for ensuring the health needs and rights of the people are being met at the community level. This would alsofoster community ownership and legitimacy of health reforms [39]. Child mortality remains one of the primary public health challenges faced by the region and consequently programs related to the prevention and reduction of child mortality require a combined effort at all levels of goverment. Despite the remarkable progress made by three EAC countries (Rwanda, Uganda, Tanzania) to achieve the MDG 4 (Table 1), there is still a lot to be done in order to reduce preventable child mortality among these countries and across the EAC region as a whole. Table 1 summarizes the EAC context including population size, economic and key health indicators. With a median age ranging from 15.9 years to 19.6 years, the EAC has one of the youngest populations globally (Table 1). Similarly, the region has one of the world highest birth rates (Table 1).

**Table 1:** Characteristics of the EAC countries included in our analysis

Characteristics	Burundi	Kenya	Rwanda	Uganda	Tanzania
Population, 2018	11,129,204	50,644,314	12,429,546	43,921,666	58,650,994
Population density per Km^2^	437	90	507	222	67
Median age, years	17.6	19.2	19.6	15.9	17.4
Fertility rate	5.91	4.03	4.11	5.82	5.17
Birth rate (births/1000 population), 2017	41	24	31	43	36
GDP per capita (current US$), 2016	285.7	1455.4	702.8	580.4	877.5
Health spending per capita (current US$), 2014	22	78	52	52	52
OOP expenditure, % healthcare expending, 2014	44.5	67.4	45.4	54.6	43.3
Health expenditure, public (% of government expenditure), 2014	13.2	12.8	9.9	11.0	12.3
External resources for health (% of total expenditure on health), 2014	50.3	27.5	46.2	35.5 (2013)	35.9
Physician / 1000 population, 2015	0.026	0.204	0.064	0.093	0.022
Density of nursing and midwifery personnel, 1000 population, 2014	0.176 (2004)	1.582	0.832	0.648	0.416
Life expectancy at birth, years, 2015	57.1	62.2	64.7	59.2	65.5
Ranking HDI index, 2015	184	146	159	163	151
Under-five mortality rate per 1000 live births, (2015)	82	49	42	55	49
Neonatal mortality rate/1000 live births	29	22	19	19	19
Infant mortality rate/1000 live births	54	36	31	38	35
Stillbirth rate per 1000 live births	28	22	23	25	26
Maternal mortality ratio (maternal deaths per 100,000 live births), 2015	712	510	290	343	398
Progress towards MDG					
Achieved MDG4	No	No	Yes	Yes	Yes
Achieved MDG5	No	No	Yes	No	No

GDP, gross domestic product; OOP, out of pocket; HDI, health development index; MDG, millennium development goal; US$, United States dollar; EAC, East African Community. Data presented in Table 1 were abstracted from various publications [26, 40–45]

### Selection of community level interventions

Drawing on prior research [37, 38, 46, 47], we identified 10 preventive and curative CLIs that have been reported to reduce child mortality: Breastfeeding promotion, complementary feeding, vitamin A supplementation, Zinc for treatment of diarrhea, hand washing with soap, hygienic disposal of children’s stools, oral rehydration solution (ORS), oral antibiotics for treatment of pneumonia, treatment for moderate acute malnutrition (MAM) and prevention of malaria using insecticide-treated nets and indoor residual spraying (ITN/IRS). These interventions can be classified into three categories:

- Prevention (prevent diseases/deaths), for example, hand washing with soap and hygienic disposal of children’s stools, ITN/IRS;
- Nutrition, for example, breastfeeding and complementary feeding; and
- Curative/treatments (stop deaths from occurring), for example, ORS for diarrhea and oral antibiotics for treatment of pneumonia.

Each of these interventions has impact on specific cause(s) of death and/or risk factors [37, 38, 46–51]. For example, vitamin A supplementation, Zinc for treatment of diarrhea, hand washing with soap, hygienic disposal of children’s stools, and ORS interventions reduce child mortality by decreasing diarrhea. Oral antibiotics for treatment of pneumonia intervention reduce child mortality by decreasing deaths due to pneumonia, while ITN/IRS prevent malaria and related deaths. Interventions that have impact on risk factors for disease (for example, breastfeeding and complementary feeding) affect multiple causes of child mortality by modifying the probability of death due to specific causes of death. For example, interventions that reduces stunting and wasting will also indirectly reduce the probability of dying of diarrhea, pneumonia and malaria.

We focused on interventions that can be delivered at community level by CHWs. Nine of the 10 CLIs that we selected are delivered at community level at least 50% (Table 2). We retrieved data on the percent of each interventions per delivery channel from Lives Saved Tool (described below) and, our modeling exercise assumed that the delivery channel for each intervention would remain constant over the study horizon. Similarly, it is assumed that variations in intervention coverage drive mortality changes, and the impacts on mortality of distal factors (for example, socioeconomic status) are mediated by changes in intervention coverage [49–52].

**Table 2:** Percent of each intervention delivered at each level of healthcare delivery channels across EAC

Interventions^a^	Community	Outreach	Clinic
1. Breastfeeding promotion	40	10	50
2. Complementary feeding	50	0	50
3. Vitamin A supplementation	50	0	50
4. Hand washing with soap	100	0	0
5. Hygienic disposal of children’s stools	100	0	0
6. ITN/IRS	50	50	0
7. Oral rehydration solution	50	0	50
8. Zinc supplementation for diarrhea	50	0	50
9. Oral antibiotics for the treatment of pneumonia	50	0	50
10. Treatment for moderate malnutrition (MAM)	50	0	50

^a^Included interventions that are offered at community at 40% or more

Breastfeeding promotion (exclusive breastfeeding 1-5 months). *ITN/IRS* insecticide-treated bed nets (*ITNs*) and indoor residual spraying (*IRS*); *EAC* East African Community. Source: Lives Saved Tool

### Modelling approach

**Fig. 1:**
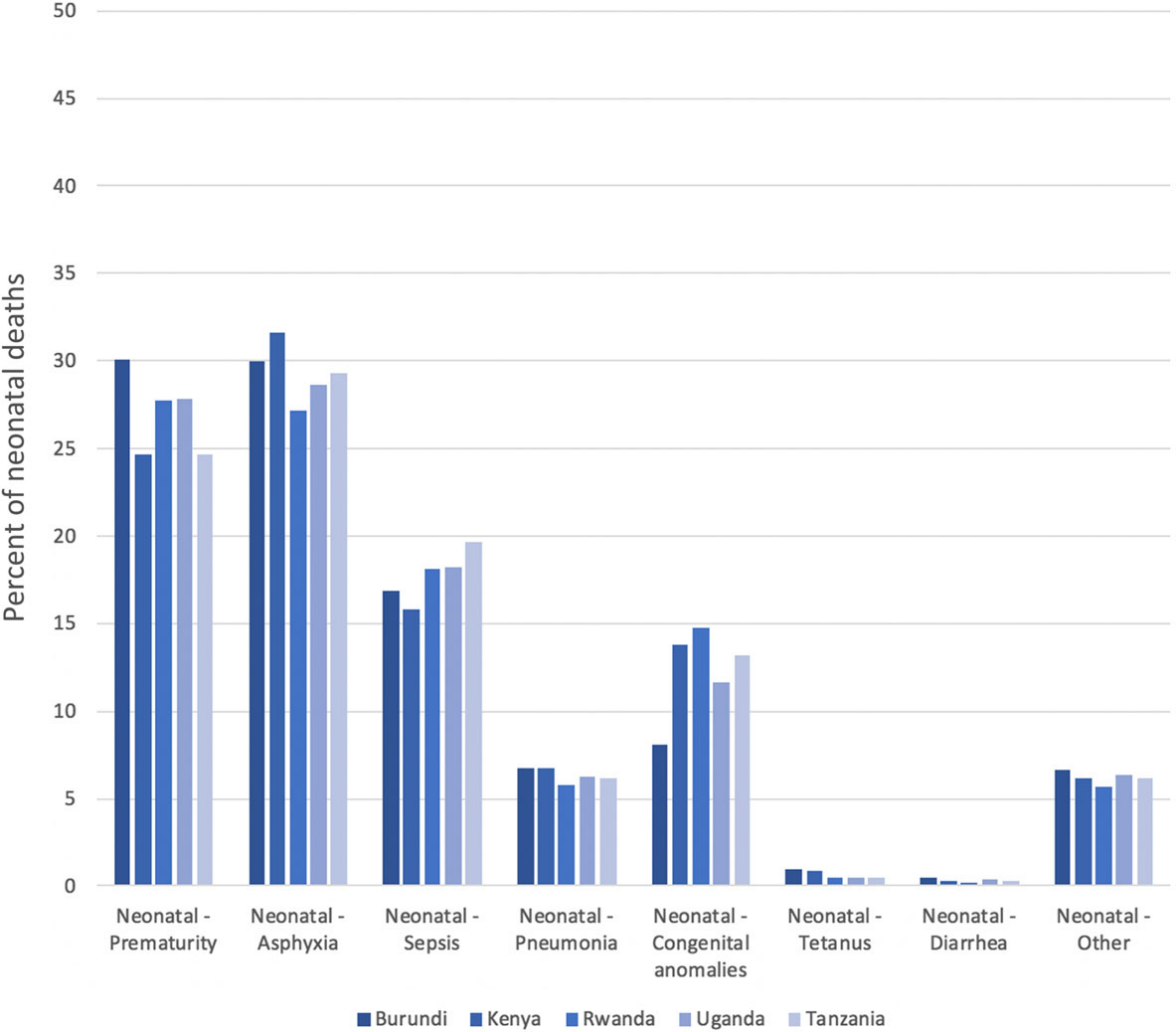
Percent of neonatal deaths by proximate causes across East African Community (2014/2015). Source: Lives Saved Tool

**Fig. 2:**
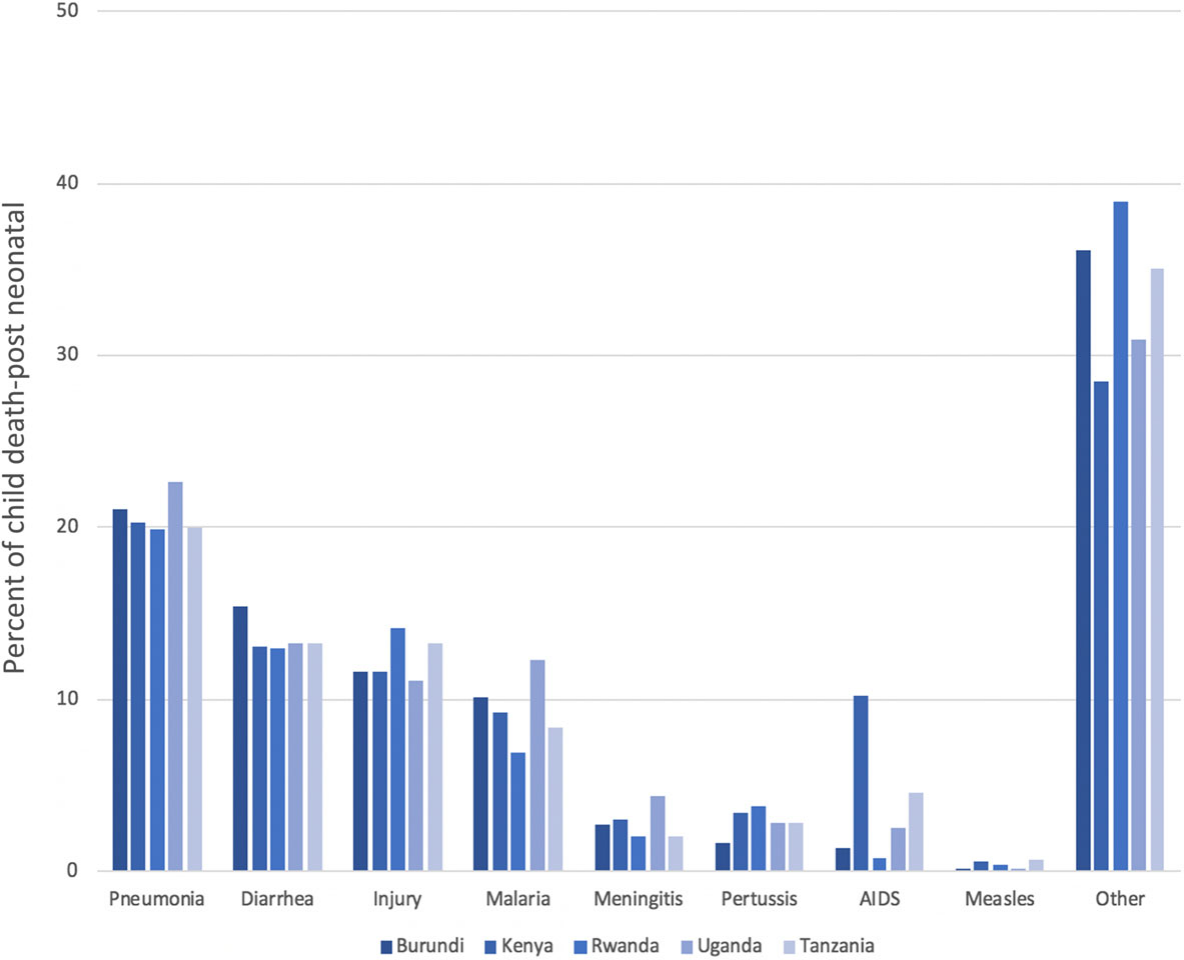
Percent of child death-post neonatal by proximate causes across East African Community (2014/2015). Source: Lives Saved Tool. While the details for ‘Other’ in the Fig. 2 was not provided in LiST, drawing on existing literature of global burden of diseases, injuries and risk factors, we believe that this section would include malnutrition, congenital anomalies, drowning, and foreign bodies [58]

We used the Lives Saved Tool (LiST) [53, 54] – one of the modules in the Spectrum software package – to model the number of deaths among children younger than five years that could be prevented across EAC as a result of expanding proven effective CLIs (change in coverage), while accounting for EAC country specific health status (Table 1) and distribution of cause-specific mortality (Figs. 1 and 2). LiST has been used widely in lower- and middle-income countries (LMICs) to estimate the potential impact and cost of expanding maternal, newborn and child health interventions across the continuum of care [37, 38, 55–57].

To make the projections, LiST employs a linear deterministic model and links with other modules (e.g., Family Planning module, AIDS Impact module and Demographic Projections module) available in the Spectrum package [53]. Our LiST model input include estimates of intervention effects and intervention coverage – defined as “the proportion of women and children in need of life–saving intervention who actually receive it” [37]. The model output was the number of deaths prevented disaggregated by each CLI. Estimates of the effects of interventions on cause specific child mortality were generated using the Child Health Epidemiology Reference Group intervention review process that draws on Cochrane Collaboration and the Working Group for Grading of Recommendations Assessment, Development and Evaluation (GRADE) [59]. The baseline population level coverage data for each intervention were derived from the most recent nationally representative surveys including demographic and health survey (DHS) and world population prospects (WPP) [37, 53].

Using LiST, we modeled the impact on under-five child mortality of scaling up the 10 CLIs from baseline coverage (2016) to ideal coverage (99%) by 2030 (Table 3). To estimate the impact under the ideal scale up scenario, we increased the coverage only for the 10 interventions that can be delivered by CHWs at the community level (Table 3), while holding all baseline population level coverage for other interventions in LiST module constant. We increased the coverage of our target interventions gradually using linear interpolation from 2016 to 2030 (i.e., study time horizon) (Table 3). We selected the study time horizon to cover the period post MDG era through the end of SGD era. To estimate the counterfactual (what would happen under business as usual (BAU) scenario), we forecasted coverage of the 10 interventions from 2016 to 2030 based upon existing trends in coverage for these interventions from 2010 to 2016 (7 years) using exponential smoothing methods and adjusted for seasonality as appropriate. We then calculated (and report in the results) number of deaths that could be prevented by ideal scale up of the 10 CLIs relative to scale up under business as usual scenario (Table 4).

**Table 3:** Baseline coverage and percent scale-up for community level interventions across EAC

Interventions	Baseline coverage, year 2016					Mean baseline coverage across EAC	Target scale-up by year 2030 across the five EAC countries
	Burundi	Kenya	Rwanda	Uganda	Tanzania		
Breastfeeding promotion^a^	80.84	58.71	86.24	61.31	52.11	67.84	99.0
Complementary feeding^b^	19.3	40.90	30.07	30.24	26.03	29.30	99.0
Vitamin A supplementation	78.0	41.0	96.0	66.0	89.0	74.0	99.0
Hand washing with soap	5.8	49.70	37.60	27.42	51.85	34.47	99.0
Hygienic disposal of children’s stools	73.7	83.07	88.11	76.28	75.24	79.28	99.0
ITN/IRS	46.8	62.50	82.93	80.83	72.06	69.02	99.0
Zinc for treatment of diarrhea	15.0	8.12	0.2	40.26	17.45	16.20	99.0
ORS	37.6	53.81	27.45	46.71	44.75	42.06	99.0
Oral antibiotics for the treatment of pneumonia	58.5	65.74	53.94	71.27	55.44	60.97	99.0
Treatment for moderate malnutrition (MAM)	0.0	0.0	0.0	0.0	0.0	0.0	99.0

^a^Excluding breastfeeding; ^b^Supplementary feeding and education; *CLIs* community-level interventions, *EAC* East African Community, *ORS* oral rehydration solution, *ITN/IRS* insecticide-treated bed nets (*ITNs*) and indoor residual spraying (*IRS*)

**Table 4:** Number of deaths averted by target year (2030) by intervention under ideal coverage scenario relative to BAU scenario

Interventions	Burundi	Kenya	Rwanda	Uganda	Tanzania
Breastfeeding practices due to promotion	─	324	55	1346	1261
Vitamin A supplementation	368	992	─	135	361
Hand washing with soap	1579	1374	158	3374	2984
ITN/IRS - Households protected from malaria	1261	─	197	─	─
Complementary feeding	394	231	103	371	789
ORS - oral rehydration solution	2365	1968	1479	4988	8529
Zinc for treatment of diarrhea	157	913	433	─	901
Oral antibiotics for pneumonia	2824	2552	1567	6845	10,455
MAM - treatment for moderate acute malnutrition	1154	1908	362	3554	3634
Total (sensitivity bound)*	10,102 (8210–11,870)	10,262 (7831–12,619)	4354 (3678–4958)	20,613 (16049–25,162)	28,914 (23300–34,002)

*ITN/IRS* insecticide-treated bed nets (*ITNs*) and indoor residual spraying (*IRS*), *BAU* business as usual. *Sensitivity bounds were derived from sensitivity analyses that estimated effects of interventions based upon the highest level of effectiveness reported for all interventions (upper bound) relative to the lowest levels of effectiveness (lower bound). An em dash (─) indicates that the item is not applicable, or the value is zero, because the coverage under BAU scenario reached 99% by 2030, which is equivalent to the coverage under the ideal scale up scenario

**Fig. 3:**
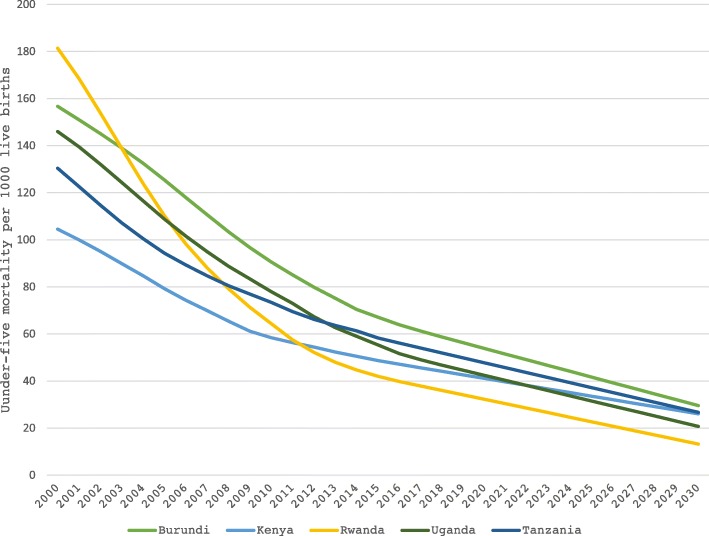
Reported and forecasted trends in under-five mortality across EAC (UNICEF reported estimates, 2000–2017, and forecasted estimates, 2018–2030). We forecasted under-five mortality trends in EAC from 2018 to 2030 using UNICEF reported under-five mortality from 2000 to 2017 and autoregressive integrated moving average time series approach. Based on our forecasted estimates, Rwanda and Uganda would meet the SDG target for under-five mortality of at least as low as 25 per 1000 live births

For intervention coverage where the existing trends were decreasing in the period of 2010–2016, forecasting the coverage from 2016 to 2030 would have led to considerably lower coverage by 2030 under BAU scenario, thus overestimating the number of deaths prevented under ideal scale up scenario relative to BAU scenario. Given ongoing emphasis on increasing coverage community level interventions to help LMICs achieve universal health coverage by 2030, it is unlikely that the decreasing trend in coverage reported for some interventions (from 2010 to 2016) would continue to 2030. As such, we used a more conservative approach by using mean coverage from the existing trends over 7 years (2010–2016) instead of the decreasing forecasted values. We assumed the percent delivery of each CLI at various delivery channels constant throughout the time horizon (Table 3). Using autoregressive integrated moving average (ARIMA) time series approach and reported under-five mortality from 2000 to 2017, we forecasted under-five mortality trends in EAC up to 2030 (Fig. 3). We used Spectrum software v5.753 (https://www.livessavedtool.org/listspectrum) and R software 3.4.4 for all analyses [60].

## Results

Our analyses included five EAC countries with a combined population of approximately 176,775,724 (Table 1). The leading causes of under-five mortality across the EAC region are summarized in the Figs. 1 and 2. Prematurity, birth asphyxia and sepsis are the top three causes of neonatal mortality across the EAC region (Fig. 1). Pneumonia and diarrhea are the top two leading causes of (post-neonatal) child mortality (excluding other causes) in most EAC region (Fig. 2). Malaria accounts for up to 10% of child death in most EAC countries. These three conditions (pneumonia, diarrhea and malaria) can been treated and / or prevented at the community level by community health workers trained to provide such interventions. Other causes of neonatal and post-neonatal death can be found elsewhere [58]. Based on our forecasted estimates (Fig. 3), of the five EAC countries, Rwanda and Uganda would meet the SDG target for under-five mortality of at least as low as 25 per 1000 live births.

The baseline coverage of the 10 interventions is not homogenous across the region (Table 3). For example, the baseline coverage of breastfeeding promotion is higher in Rwanda and Burundi compared to the rest of EAC countries (Table 3). Similarly, vitamin A supplementation baseline coverage seems to be higher in Rwanda and Tanzania and lower in Kenya. The mean coverage of oral antibiotics for the treatment of pneumonia for EAC is 60.9%, with the highest rate reported in Uganda (71.3%) followed by Kenya (65.7%). Likewise, the mean coverage of ITN/IRS is 69.0%, with the highest rate reported in Rwanda (82.9%) followed by Uganda (80.8%) (Table 3). The mean coverage of ORS is overall low (42.1%), with the highest rate reported in Kenya (53.8%) and lowest in Rwanda (27.5%). Zinc supplementation and MAM coverage are extremely low across the region; while the baseline coverage of hygienic disposal of children’s stools is higher across the region (mean coverage: 79.3%).

The pre-existing coverage for most of interventions we analyzed was increasing from 2010 to 2016 and, as such, the forecasted coverage for some interventions reached 99% under BAU coverage. These include breastfeeding promotion in Burundi, ITN/IRS in Kenya, vitamin A supplementation in Rwanda, Zinc supplementation for diarrhea and ITN/IRS in Uganda, and ITN/IRS in Tanzania. However, at the same time the pre-existing coverage was decreasing for some interventions. In Burundi, the pre-existing coverage was decreasing for Vitamin A supplementation, handwashing with soap, hygienic disposal of children’s stools, ORS, and ITN/IRS. In Kenya, the pre-existing coverage was decreasing for vitamin A supplementation. In Rwanda, the pre-existing coverage was decreasing for ORS. In Uganda, the pre-existing coverage was decreasing for hygienic disposal of children’s stools and oral antibiotics for the treatment of pneumonia. In Tanzania, the pre-existing coverage was decreasing for complementary feeding, vitamin A supplementation, and oral antibiotics for the treatment of pneumonia.

**Fig. 4:**
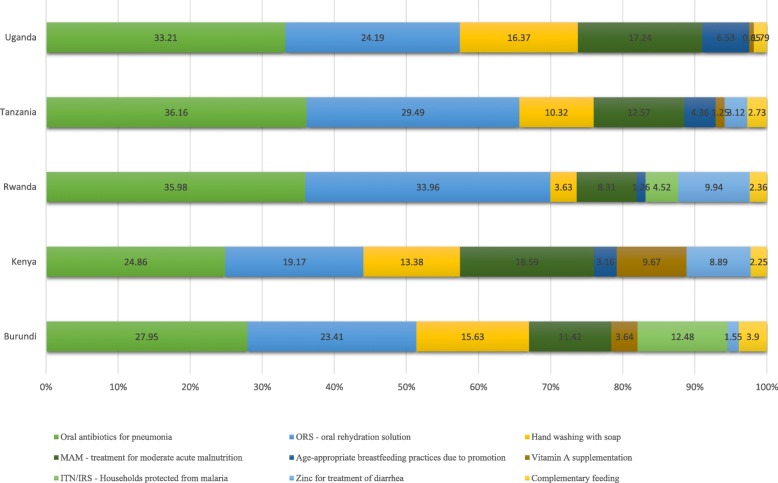
Percent of deaths averted by 2030 by intervention under ideal coverage scenario relative to business as usual coverage scenario

Our analysis suggests that, compared to BAU coverage scenario, ideal scale-up of the 10 interventions could prevent approximately 74,200 (sensitivity bounds 59,068–88,611) child deaths by 2030 including 10,100 (8210–11,870) deaths in Burundi, 10,300 (7831–12,619) deaths in Kenya, 4350 (3678–4958) deaths in Rwanda, 20,600 (16049–25,162) deaths in Uganda, and 28,900 (23300–34,002) deaths in the United Republic of Tanzania (Table 4). Effective scale up of oral antibiotics for the treatment of pneumonia could save the highest number of lives, accounting for approximately 1/3 of all lives saved in Rwanda, Tanzania and Uganda, and about ¼ in the rest of EAC countries (Fig. 4). Oral rehydration solution for treatment of diarrhea is the top 2 live saving CLI, accounting for at least about a fifth of all lives saved across EAC countries (Fig. 4). Overall, the top four interventions (oral antibiotics for pneumonia, ORS, hand washing with soap, and treatment for MAM) account for over 75.0% of all deaths prevented in each EAC country: 78.4% in Burundi, 76.0% in Kenya, 81.8% in Rwanda, 91.0% in Uganda and 88.5% in Tanzania. The remaining five CLIs (breastfeeding promotion, ITN/IRS, complementary feeding, vitamin A supplementation, hygienic disposal of children’s stools, and Zinc supplementation for diarrhea) could account for just about a fifth of all lives saved in Burundi, Kenya and Rwanda, and about one in ten of lives saved in Uganda and Tanzania (Fig. 4).

## Discussion

Our study aimed to estimate the potential impact of expanding various CLIs on child mortality in five EAC countries (Burundi, Kenya, Rwanda, Uganda and Tanzania). We identified 10 preventive and curative community-level interventions that have been reported to reduce child mortality: Breastfeeding promotion, complementary feeding, vitamin A supplementation, Zinc for treatment of diarrhea, hand washing with soap, hygienic disposal of children’s stools, oral rehydration solution (ORS), oral antibiotics for treatment of pneumonia, MAM – treatment for moderate acute malnutrition, and ITN/IRS – households protected from malaria. We found that, compared to BAU coverage scenario, ideal scale-up of the 10 interventions could prevent approximately 74,200 child deaths by 2030 including 10,100 deaths in Burundi, 10,300 deaths in Kenya, 4350 deaths in Rwanda, 20,600 deaths in Uganda, and 28,900 deaths in Tanzania. The top four interventions (oral antibiotics for pneumonia, ORS, hand washing with soap, and treatment for MAM) account for over 75.0% of all deaths prevented in each EAC country: 78.4% in Burundi, 76.0% in Kenya, 81.8% in Rwanda, 91.0% in Uganda and 88.5% in Tanzania.

Universal healthcare as one of the SDGs offers an avenue through which the top four interventions and other interventions could be scaled-up through health campaign messages and basic services offered at each institutional point of care. As noted by Yamin et al. (2017), universal healthcare achievement requires a strong human rights landscape and policy frameworks to enable people to affect policy and in turn take a lead role in policy-backed implementation of universal healthcare [39]. Countries with more stable political landscape could arguably make better progress towards universal healthcare than countries under unstable political landscapes.

Consistent with prior research, [61] our forecasted estimates suggest that, of the five EAC countries, only Rwanda and Uganda would meet the SDG target for under-five mortality of at least as low as 25 per 1000 live births, based on pre-existing trends in child mortality. For the rest of EAC countries, further decline in child mortality would be required to meet the child health related SDG target. Different studies conducted in LIMCs have highlighted the role of implementing CLIs in the prevention of child mortality. Studies have also shown the role of involving CHWs to providing CLIs in the endeavor to ending preventable child mortality. Nonetheless, despite the remarkable results of CHWs contributions, health systems in many LMICs and particularly in the EAC region have not yet scaled CHWs programs up to the countries’ ideal levels. In order to achieve desirable results in child mortality reduction using available resources in the EAC countries of focus, a closer attention should be put on strengthening CLIs along with CHWs programs. The fact that the top four interventions in our findings (oral antibiotics for pneumonia, ORS, treatment for MAM, and hand washing with soap) could account for over 75% of all deaths prevented in each EAC countries makes a strong argument for the impact these low-cost interventions have when implemented and scaled-up efficiently. It is also noteworthy that were these interventions to be implemented as part of basic services offered at outreach centers, their impact on death prevention could increase, especially given that maternal education has been shown to be a key driver in the successful implementation and uptake of these interventions, particularly in Kenya, but also elsewhere in the EAC region [62].

As discussed earlier, the potential impact of scaling up the 10 interventions appears to vary across EAC countries. This can be explained, in part, by heterogeneity in pre-existing trend in coverage for these interventions from 2010 to 2016 and, ultimately, the related forecasted coverage through 2030 under BAU coverage scenario. For example, the forecasted coverage for ITN/IRS in Kenya, Uganda and Tanzania reached 99% by 2030. As such, given the coverage of ITN/IRS under BAU scenario is equivalent to ideal coverage scenario, there was no enough room for improvement to save additional lives beyond those saved under BAU scenario. Conversely, in countries where the pre-existing trend in certain intervention coverage was decreasing and / or low, scaling up these interventions appears to account for a higher proportion of lives saved in the same countries relative to the rest of EAC. Similarly, the baseline coverage of hygienic disposal of children’s stools is quite high across the EAC region (79%). Therefore, its effective scale up (99%) doesn’t appear to save more lives given there was little room for scale-up compared to hand washing with soap, with a baseline coverage of 34%.

There are still gaps in research on the contributions of CLIs on the reduction and prevention of child mortality among EAC countries. Findings from studies done in settings with similar resources have been generalized with the aim to promote child health initiatives at the community level [14, 63]. Even though such replications have shown success in reducing child mortality, further research is needed, in order to look at each country’s specific context of child health and under-five mortality. A number of determinants of health as well as available inequalities and inequities in the country’s different systems all impact the outcome of child health. For example, the availability of services in rural vs urban settings and other socio-economic factors such as education, income, community and family support are associated with effectiveness of CLIs [8]. In addition, channels of implementation of such interventions need to be identified in order to evaluate their success and sustainability. It has been reported that CHWs in different EAC countries have not received attention until the last two decades. Even where they did, their programs have not yet been fully integrated in the country’s health systems. As a result, there are still both inequalities and inequities in child health outcomes within countries, and differences are observed among areas where CHWs operate when compared to where they do not. Among the five EAC countries, Rwanda is the only country where the CHWs are part of the health system. CHWs provide promotional, preventive and curative health care services for maternal and child health. Since their involvement in the health system, remarkable results in child mortality reduction have been observed [15–18].

There are many reasons explaining why different countries have not yet been able to integrate CHWs in their health systems. In most cases, government have failed to secure funding for the sustainability of CHWs. In such cases, CHWs programs are partially implemented by government or different NGOs to implement specific interventions and these would slow down or stop once funding finished. In addition, not having specific roles for CHWs and increasing their workload have been reported to slow down the progress towards the implementation of CLIs. Even though CHWs have been involved in the primary health care for more than fifty years in some countries, it is still unclear what exactly their roles and responsibilities for child health are, where their responsibilities start and end, and what responsibilities governments have to support them [64]. In most cases, CHWs are members of the community who are selected by their own communities to champion selected health initiatives through the provision of promotional and preventive services [65]. For the most part, CHWs are volunteers and do not have or receive any formal health care training [66].

CHWs are community volunteers with no formal education or child health training and have considerable workloads, which always pose challenges regarding what type of interventions to provide and to whom (rural vs urban, poor vs wealthy, educated vs uneducated). Policy makers would need to revisit and reform these programs in order to achieve sustainable and positive child health outcomes at all levels. Sing and Sachs (2013) highlight that if CHWs are considered an integral part of the child health system, they benefit from the provision of better training and supervision and are able to contribute to the health reporting and feedback systems [64]. In Rwanda, community level programs appeared to be associated with a significant increase in coverage of maternal and child health services when CHWs benefited from additional support including regular training, supervision and resources [15, 21]. Using a theory of change approach, a recent evaluation of Rwanda community health program found that the program has been successful in delivering targeted essential interventions at scale [67]. Similarly, Scott and colleagues (2018) systematic review of existing 122 reviews suggest several factors associated with positive CHW program outcomes including supportive supervision, community embeddedness, continuing education, and adequate resources (logistical support and supplies), but they also highlighted areas where there are significant evidence gaps to inform the global research agenda for community health systems [68]. To achieve ideal coverage of CLIs, the EAC countries would need to increase CHWs while also affording them similar additional support.

Like any modelling studies, there are limitations that should be acknowledged. First, while our LiST model provided country specific estimates and considered country specific underlying health status and cause specific mortality, real-world context such as CLI implementation fidelity across EAC region could not be accounted for. The modelling approaches assumed perfect fidelity for CLIs, which is not realistic given that interventions are rarely implemented with perfect fidelity in real-world settings [69, 70]. As such, we could have overestimated the impact of various CLIs we studied. Second, we assumed the percent delivery of each community level intervention at various delivery channels constant throughout the time horizon (Table 3). However, it is possible that the percent delivery channel would change over the study horizon. Similarly, while our LiST model employed linear interpolation to estimate the impact of various CLIs over the study horizon, it is likely that the scale up level would not follow the linear increment assumed in the LiST model.

In conclusion, scaling up interventions that can be delivered at community level by community health workers can contribute to substantial reduction of child mortality in EAC and could help EAC region achieve the child health related SDG target. Our findings suggest the top four CLIs that account for more than three-quarters of all deaths prevented across EAC countries. Going forward, costs of scaling up each intervention will be estimated to guide policy decisions including resource allocations in EAC countries.

## References

[1] Chao F, You D, Pedersen J, Hug L, Alkema LJTLGH (2018). National and regional under-5 mortality rate by economic status for low-income and middle-income countries: a systematic assessment.

[2] Hug L, Sharrow D, You D (2017). Levels & trends in child mortality: report 2017. Estimates developed by the UN inter-agency Group for Child Mortality Estimation.

[3] World Health Organization. Global health estimates 2016: disease burden by cause, age, sex, by country and by region, 2000–2016. Geneva; 2018.

[4] You D, Hug L, Ejdemyr S, Beise J (2015). Levels and trends in child mortality. Estimates developed by the UN inter-agency Group for Child Mortality Estimation (IGME).

[5] Liu L, Oza S, Hogan D, Perin J, Rudan I, Lawn JE, et al. Global, regional, and national causes of child mortality in 2000–13, with projections to inform post-2015 priorities: an updated systematic analysis. 2015;385(9966):430–40. 10.1016/S0140-6736(14)61698-6 25280870

[6] You D, Hug L, Ejdemyr S, Idele P, Hogan D, Mathers C, et al. Global, regional, and national levels and trends in under-5 mortality between 1990 and 2015, with scenario-based projections to 2030: a systematic analysis by the UN Inter-agency Group for Child Mortality Estimation. 2015;386(10010):2275–86. 10.1016/S0140-6736(15)00120-8 26361942

[7] Assembly UG. Transforming our world: the 2030 agenda for sustainable development. 2015. Contract no.: 1.

[8] Rutherford ME, Mulholland K (2010). Hill PCJTm, health i. How access to health care relates to under-five mortality in sub-Saharan Africa: systematic review.

[9] Mundial B. World development report 1993; investing in health: Oxford University Press; 1993.

[10] Lewin S, Munabi-Babigumira S, Glenton C, Daniels K, Bosch-Capblanch X, van Wyk BE, et al. Lay health workers in primary and community health care for maternal and child health and the management of infectious diseases. Cochrane Libr. 2010. 10.1002/14651858.CD004015.pub3 PMC6485809 20238326

[11] Ballard M, Madore A, al. e. Concept note: Community Health Workers2018.

[12] Fulton BD, Scheffler RM, Sparkes SP, Auh EY, Vujicic M (2011). Soucat AJHrfh. Health workforce skill mix and task shifting in low income countries: a review of recent evidence.

[13] Haines A, Sanders D, Lehmann U, Rowe AK, Lawn JE, Jan S (2007). Achieving child survival goals: potential contribution of community health workers. Lancet.

[14] Christopher JB, Le May A, Lewin S, Ross DA (2011). Thirty years after Alma-Ata: a systematic review of the impact of community health workers delivering curative interventions against malaria, pneumonia and diarrhoea on child mortality and morbidity in sub-Saharan Africa. Hum Resour Health.

[15] Musabyimana A, Ruton H, Gaju E, Berhe A, Grépin KA, Ngenzi J, et al. Assessing the perspectives of users and beneficiaries of a community health worker mHealth tracking system for mothers and children in Rwanda. 2018;13(6):e0198725. 10.1371/journal.pone.0198725 PMC5991741 29879186

[16] Abbott P, Sapsford R, Binagwaho AJWD. Learning from success: how Rwanda achieved the millennium development goals for health. 2017;92:103–16.

[17] Condo J, Mugeni C, Naughton B, Hall K, Tuazon MA, Omwega A (2014). Rwanda’s evolving community health worker system: a qualitative assessment of client and provider. perspectives..

[18] Tuyisenge G, Hategeka C, Kasine Y, Luginaah I, Cechetto D, Rulisa SJW (2018). Mothers’ perceptions and experiences of using maternal health care services in Rwanda.

[19] Hitimana M (2018). Abajyanama b’ubuzima bazamuye ubwitabire bw’ababoneza urubyaro, bashobora no gutera urushinge. Igihe.

[20] Hategeka C, Ruton H, Law MRJGHR (2019). Policy. Effect of a community health worker mHealth monitoring system on uptake of maternal and newborn health services in Rwanda.

[21] Ruton H, Musabyimana A, Gaju E, Berhe A, Grépin KA, Ngenzi J, et al. The impact of an mHealth monitoring system on health care utilization by mothers and children: an evaluation using routine health information in Rwanda. 2018;33(8):920–7. 10.1093/heapol/czy066 PMC6172419 30169638

[22] Ministère à la Présidence chargé de la Bonne Gouvernance et du Plan [Burundi] (MPBGP), Ministère de la Santé Publique et de la Lutte contre le Sida [Burundi] (MSPLS), Institut de Statistiques et d’Études Économiques du Burundi (ISTEEBU), ICF. Troisième Enquête Démographique et de Santé. Bujumbura, Burundi; 2017.

[23] Worldwide C (2018). Community health workers’ role in improving child health in Burundi.

[24] IntraHealth. Uganda Takes Major Steps to Professionalize Community Health Workforce 2018 [cited 2018 November 18, 2018]. Available from: https://www.intrahealth.org/news/uganda-takes-major-steps-professionalize-community-health-workforce.

[25] Namazzi G, Okuga M, Tetui M, Muhumuza Kananura R, Kakaire A, Namutamba S (2017). Working with community health workers to improve maternal and newborn health outcomes: implementation and scale-up lessons from eastern Uganda. J Global health action.

[26] World Health Organization. Global Health Observatory (GHO) data: health workforce 2017 [April 06, 2018]. Available from: http://www.who.int/gho/health_workforce/en/.

[27] WHOJR. Under-five mortality data by country [data file]. 2015.

[28] Project CHW-LA. CHW-LAP supporting the implementation of a community health worker cadre in Tanzania 2018 [cited 2018 November 18, 2018]. Available from: https://chw-lap.muhas.ac.tz/index.php/history-of-chws-tanzania.

[29] Vision W (2015). Tanzania’s community health workers.

[30] Communities AP. Country profile: Kenya community health programs. Arlington, VA; 2013.

[31] Ngugi AK, Nyaga LW, Lakhani A, Agoi F, Hanselman M, Lugogo G (2018). Prevalence, incidence and predictors of volunteer community health worker attrition in Kwale County, Kenya. J BMJ global health.

[32] Shakir FK (2010). Community health worker programs: a review of recent literature.

[33] Khanal S, Jaganath Sharma VSG, Dawson P, Houston R, Khadka N, Yengden BJJoh, population,, et al. Community health workers can identify and manage possible infections in neonates and young infants: MINI—a model from Nepal 2011;29(3):255. 10.3329/jhpn.v29i3.7873 PMC3131126 21766561

[34] Neupane D, Dawson P, Houston R, Dhakal L, Sharma J, Gargi K, et al. Lower mortality is observed among low birth weight young infants who have received home-based care by female community health volunteers in rural Nepal. 2017;17(1):218. 10.1186/s12884-017-1355-z PMC5504681 28697728

[35] Abbey M, Chinbuah MA, Gyapong M, Bartholomew LK, BJBph v d B (2016). Community perceptions and practices of treatment seeking for childhood pneumonia: a mixed methods study in a rural district. Ghana..

[36] Abbey M, Bartholomew LK, Chinbuah MA, Gyapong M (2017). Gyapong JO, van den Borne BJBph. Development of a theory and evidence-based program to promote community treatment of fevers in children under five in a rural district in Southern Ghana: An intervention mapping approach.

[37] Chou VB, Friberg IK, Christian M, Walker N, Perry HB. Expanding the population coverage of evidence–based interventions with community health workers to save the lives of mothers and children: an analysis of potential global impact using the lives saved tool (LiST). J Glob Health. 2017;7(2). 10.7189/jogh.07.020401 PMC5592116 28959436

[38] Lungiswa L, Lumbwe LC, Aviva AT, Karen KH (2017). Modelling the cost of community interventions to reduce child mortality in South Africa using the lives saved tool (LiST). BMJ Open.

[39] Yamin AE, AJBih M (2017). Rights h. realizing universal health coverage in East Africa: the relevance of. human rights.

[40] Worldometers. Population: eastern Africa 2018 [April 06, 2018]. Available from: http://www.worldometers.info/population/africa/eastern-africa/

[41] World Bank. World Bank national accounts data, and OECD National Accounts data files: GDP per capita (current US$) 2018 [April 06, 2018]. Available from: https://data.worldbank.org/indicator/NY.GDP.PCAP.CD?locations=KE.

[42] Bank W. Health spending per capita 2018 [April 06, 2018]. Available from: https://data.worldbank.org/indicator/SH.XPD.PCAP

[43] United nations development programme. Human Development Index Report 2017 [April 06, 2018]. Available from: http://hdr.undp.org/en/composite/HDI

[44] UNICEF, World Health Organization (2015). A decade of tracking Progress for maternal. Newborn and child survival: the 2015 report.

[45] Central Intelligence Agency. World Factbook Title 2018 [September 12, 2018]. Available from: https://www.cia.gov/library/publications/the-world-factbook/.

[46] Black R, Laxminarayan R, Temmerman M, Walker N. Disease control priorities, (Volume 2). In: Reproductive, maternal, newborn, and child health: The World Bank; 2016. 27227205

[47] Requejo JH, Bhutta ZA (2015). The post-2015 agenda: staying the course in maternal and child survival. Arch Dis Child.

[48] Bhutta ZA, Das JK, Walker N, Rizvi A, Campbell H, Rudan I, et al. Interventions to address deaths from childhood pneumonia and diarrhoea equitably: what works and at what cost? 2013;381(9875):1417–29. 10.1016/S0140-6736(13)60648-0 23582723

[49] Walker CLF (2014). Walker NJBm. The Lives Saved Tool (LiST) as a model for diarrhea mortality reduction.

[50] Imdad A, ZAJBph B. Effect of preventive zinc supplementation on linear growth in children under 5 years of age in developing countries: a meta-analysis of studies for input to the lives saved tool. 2011;11(3):S22. 10.1186/1471-2458-11-S3-S22 PMC3231896 21501440

[51] Fox MJ, Martorell R, Van Den Broek N, Walker N. Assumptions and methods in the lives saved tool (LiST). BioMed Central. 2011. 10.1186/1471-2458-11-S3-I1 PMC3231881 21501425

[52] Munos M, Guiella G, Roberton T, Maïga A, Tiendrebeogo A, Tam Y, et al. Independent evaluation of the rapid scale-up program to reduce under-five mortality in Burkina Faso. 2016;94(3):584–95. 10.4269/ajtmh.15-0585 PMC4775895 26787147

[53] Walker N, Tam Y, Friberg IK. Overview of the lives saved tool (LiST). BioMed Central. 2013. 10.1186/1471-2458-13-S3-S1 PMC3847271 24564438

[54] Winfrey W, McKinnon R, JJBph S. Methods used in the lives saved tool (LiST). 2011;11(3):S32. 10.1186/1471-2458-11-S3-S32 PMC3231906 21501451

[55] Chola L, Pillay Y, Barron P, Tugendhaft A, Kerber K, Hofman K (2015). Cost and impact of scaling up interventions to save lives of mothers and children: taking South Africa closer to MDGs 4 and 5. Glob Health Action.

[56] Homer CS, Friberg IK, Dias MAB, ten Hoope-Bender P, Sandall J, Speciale AM (2014). The projected effect of scaling up midwifery. Lancet.

[57] Jo Y, Labrique AB, Lefevre AE, Mehl G, Pfaff T, Walker N (2014). Using the lives saved tool (LiST) to model mHealth impact on neonatal survival in resource-limited settings. PLoS One.

[58] Kassebaum N, Kyu HH, Zoeckler L, Olsen HE, Thomas K, Pinho C, et al. Child and adolescent health from 1990 to 2015: findings from the global burden of diseases, injuries, and risk factors 2015 study. 2017;171(6):573–92. 10.1001/jamapediatrics.2017.0250 PMC5540012 28384795

[59] Walker N, Fischer-Walker C, Bryce J, Bahl R, Cousens S (2010). epidemiology wftCRGoIEJIjo Standards for CHERG reviews of intervention effects on child survival.

[60] Team RC (2018). The R Foundation for Statistical Computing Platform.

[61] McArthur JW, Rasmussen K, Yamey GJB. How many lives are at stake? Assessing 2030 sustainable development goal trajectories for maternal and child health. 2018;360:k373. 10.1136/bmj.k373 PMC5813301 29449222

[62] Keats EC, Ngugi A, Macharia W, Akseer N, Khaemba EN, Bhatti Z, et al. Progress and priorities for reproductive, maternal, newborn, and child health in Kenya: a Countdown to 2015 country case study. 2017;5(8):e782–e95. 10.1016/S2214-109X(17)30246-2 PMC5599303 28716350

[63] Iyer HS, Chukwuma A, Mugunga JC, Manzi A, Ndayizigiye M, Anand S (2018). A comparison of health achievements in Rwanda and Burundi.

[64] Singh Prabhjot, Sachs Jeffrey D (2013). 1 million community health workers in sub-Saharan Africa by 2015. The Lancet.

[65] Lehmann U. Sanders DJTsoteop, activities, costs, organization iohoouchwGWH. Community health workers: what do we know about them. 2007:1–42.

[66] Haver Jaime, Brieger William, Zoungrana Jérémie, Ansari Nasratullah, Kagoma Jean (2015). Experiences engaging community health workers to provide maternal and newborn health services: Implementation of four programs. International Journal of Gynecology & Obstetrics.

[67] D’Aquino L, Mahieu A. 2016 Rwanda: Comprehensive Evaluation of The Community Health Program in Rwanda 20016.

[68] Scott K, Beckham S, Gross M, Pariyo G, Rao KD, Cometto G (2018). What do we know about community-based health worker programs?. A systematic review of existing reviews on community health workers.

[69] Hategekimana C, Shoveller J, Tuyisenge L, Kenyon C, Cechetto DF, Lynd LDJPo. Correlates of performance of healthcare workers in emergency, triage, assessment and treatment plus admission care (ETAT+) course in Rwanda: context matters. 2016;11(3):e0152882. 10.1371/journal.pone.0152882 PMC4816404 27030974

[70] Shoveller J, Viehbeck S, Di Ruggiero E, Greyson D, Thomson K, Knight RJCPH. A critical examination of representations of context within research on population health interventions. 2016;26(5):487–500.

